# Dogs accurately track a moving object on a screen and anticipate its destination

**DOI:** 10.1038/s41598-020-72506-5

**Published:** 2020-11-16

**Authors:** Christoph J. Völter, Sabrina Karl, Ludwig Huber

**Affiliations:** 1 Messerli Research Institute, University of Veterinary Medicine Vienna, Vienna, Austria; 2 Messerli Research Institute, Medical University of Vienna, Vienna, Austria; 3 Messerli Research Institute, University of Vienna, Vienna, Austria

**Keywords:** Attention, Animal behaviour, Psychology

## Abstract

The prediction of upcoming events is of importance not only to humans and non-human primates but also to other animals that live in complex environments with lurking threats or moving prey. In this study, we examined motion tracking and anticipatory looking in dogs in two eye-tracking experiments. In Experiment 1, we presented pet dogs (N = 14) with a video depicting how two players threw a Frisbee back and forth multiple times. The horizontal movement of the Frisbee explained a substantial amount of variance of the dogs’ horizontal eye movements. With increasing duration of the video, the dogs looked at the catcher before the Frisbee arrived. In Experiment 2, we showed the dogs (N = 12) the same video recording. This time, however, we froze and rewound parts of the video to examine how the dogs would react to surprising events (i.e., the Frisbee hovering in midair and reversing its direction). The Frisbee again captured the dogs’ attention, particularly when the video was frozen and rewound for the first time. Additionally, the dogs looked faster at the catcher when the video moved forward compared to when it was rewound. We conclude that motion tracking and anticipatory looking paradigms provide promising tools for future cognitive research with canids.

## Introduction

Dogs have become one of the most popular model species in comparative cognitive research over the last decades^1^. Most of the research to date has been based on visual stimuli. Nevertheless, the knowledge about dogs’ visual perception is limited^2,3^. Dogs’ motion perception, in particular, has received little research attention. However, it has been postulated that “dogs, like people, are much more sensitive to moving objects than they are to stationary ones”^3^ (p. 1624) mostly based on anatomical evidence.

The canine retina does not exhibit a primate *fovea centralis* but a fovea-like area within the *area centralis*^4,5^. Dogs have a higher proportion of rods compared to humans, also within the area centralis. Due to the suitability of rods for motion detection, this finding has been interpreted as evidence for high motion sensitivity in dogs^3^. Additionally, temporal information processing seems to be faster in dogs than in humans. Flicker-fusion rates (i.e., the lowest frequency in which a flickering light stimulus is perceived as a constant light source) of up to 80 Hz have been reported for dogs^6^ compared to ca. 60 Hz for humans^7,8^.

One feature of motion perception that has been studied in dogs is the detection of coherent motion. Coherent motion detection is typically examined using random-dot displays in which a certain proportion of dots moves in the same direction whereas the remaining dots move randomly. The lower this proportion of coherently moving dots the harder is the detection of coherent motion. The lowest proportion of coherently moving dots for which dogs were capable to detect coherent motion was 0.29, a lot higher than the threshold reported for adult humans of 0.05^9,10^. Thus, coherent motion detection seems to be more efficient in humans (and other mammals including macaques, cats, and seals^11–13^) than in dogs. Kanizsár and colleagues^9^ speculate that domestication might have reduced the selection pressure for accurate motion detection in dogs.

Moreover, there is evidence suggesting that dogs are sensitive to biological motion cues. Kovács and colleagues^14^ reported that dogs looked longer at a human point-light walker in lateral view (depicting the movement pattern of a walking human) compared to a control with scrambled points. However, this result could not be replicated in two subsequent studies^15,16^. Ishikawa and colleagues^16^ only found increased looking times for a human point-light walker in frontal view but not in lateral view compared to inverted control stimuli. Eatherington and colleagues^15^ did not find an increased looking time toward lateral human point-light walkers but only to upright dog point-light walkers (irrespective of scrambling of the points).

In another study, dogs interacted more often with objects that displayed a dependent movement pattern (resembling a chasing event) than with independently moving objects^17^. In a related looking time study, dogs lost interest sooner in geometric shapes with a dependent (chasing-like) movement pattern than in shapes with an independent movement pattern^18^. Even though it is unclear whether these two findings are compatible, they have been interpreted as evidence that dogs perceive moving objects as animate based on their movement patterns.

In recent years, the first eye-tracking studies have been conducted with dogs. So far, most studies examined dogs’ gaze behavior to static images^19–26^ but two studies have already used dynamic stimuli^27,28^. Correia-Caeiro and colleagues^27^ showed the dogs short videos depicting facial responses of humans and dogs and examined how dogs scanned the facial expressions. Téglás and colleagues^28^ presented dogs videos of a human turning toward one out of two identical buckets (which both had been associated with food before). Dogs were more likely to follow this directional cue with their gaze to one of the buckets if the human demonstrator had addressed them ostensively in the beginning of the video. In sum, eye-tracking studies with dogs so far mainly analyzed the proportion looking time, number of fixations, and first looks to static areas of interest but not motion tracking or anticipatory looking. In the current study, we aim to fill this gap and report the first eye-tracking data on motion tracking and anticipatory looking in dogs.

Anticipatory looking has been studied extensively in humans. Adult humans predict the location and movements of task relevant objects based on past experiences^29,30^. For example, when watching dynamic scenes such as a tennis match, human observers anticipated the location of the bounce point and fixate this point before the ball reaches this location^30,31^. In another study, human observers also anticipated forthcoming grasping sites when observing others’ actions in a block stacking task^29^. Anticipatory looking behavior has also been found in human infants, for example, in tasks aiming at goal-based action understanding^32^ and object permanence^33^. Recent evidence for anticipatory looking in non-human primates highlights the potential of this method in a comparative framework. These studies provided evidence, for instance, that non-human great apes predict upcoming events based on their long-term memory^34^ and that they anticipate actions based on attributing goals to (human) agents^35,36^.

In the current study, we examined to what extent dogs followed and anticipated movements on a screen. We showed the dogs a naturalistic scene: a video depicting how two players threw a Frisbee back and forth multiple times. We addressed the question to what extent the Frisbee movement explained the dogs’ eye movements. We were particularly interested in the question whether the dogs anticipated the destination of the moving object (here: the catcher) when they watched such a repetitive, dynamic scene (Experiment 1). In Experiment 2, we explored how the dogs would react to a potentially surprising event, the freezing and rewinding of the video. We hypothesized that dogs would anticipate the destination of the Frisbee with increasing experience. Moreover, we predicted that dogs would react to the surprising events with elevated attention.

## Results

### Experiment 1

**Figure 1:**
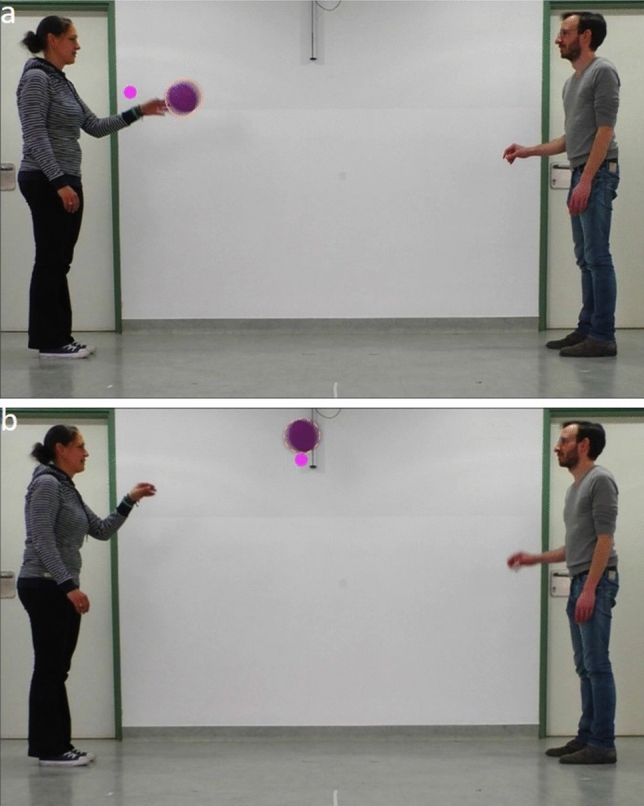
Screenshots of the video clip shown in Experiment 1 and 2. (**a**) A frame before the Frisbee arrives at the catcher is depicted. (**b**) The frame shows one of the periods when the video was frozen in Experiment 2. The circle around the Frisbee shows the dynamic area of interest. The pink dot shows the gaze location of a dog.

We presented the dogs with a video depicting how two human players threw a violet Frisbee back and forth (10 times) against a white background (see Fig. 1). In Experiment 1, we froze parts of the video at four different points in time (see Supplementary Video S1). Specifically, we froze one player (two times the left and two times the right player) while the Frisbee moved toward this player (henceforth: the catcher). We froze the catcher to examine whether the (hand) movements of the catcher could explain any anticipatory looking.

**Figure 2:**
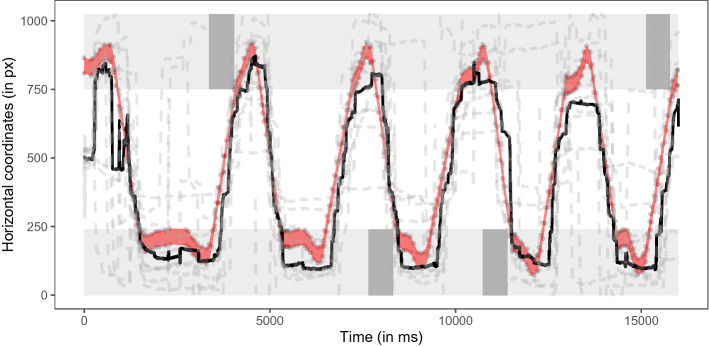
Time series plot showing dogs' horizontal gaze position across the entire 16-s video in Experiment 1. The dashed grey lines show dogs’ individual performances; the black line shows the median gaze positions; the area highlighted in red shows the position of the Frisbee. The light grey areas highlight the positions of the two players; the dark grey areas indicate when a given player was frozen before the Frisbee arrived. The figure was created in R^55^ using the R package ggplot2^58^.

For most dogs, the movement of the Frisbee explained a large part of their horizontal eye movements (median r^2^ value: 0.61; range: 0.01–0.89; see Fig. 2 and Supplementary Fig. S1 online).

**Figure 3:**
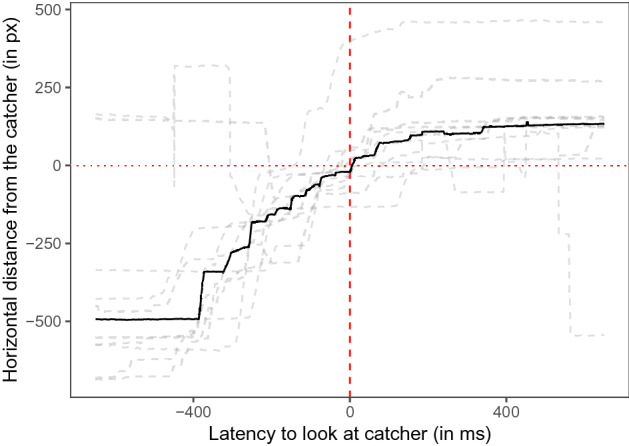
The dogs’ gaze distance from the catcher (positive values are within the catcher area of interest (AOI), negative values indicate gaze positions between the two players) as a function of the latency relative to the video frame before the Frisbee made contact with the catcher. The dashed grey lines indicate the median individual latencies; the black line indicates the mean latency (based on the individual median values). The vertical red dashed line indicates the time point right before the Frisbee made contact with the catcher. The horizontal red dotted line highlights the boundary of the catcher AOI. The figure was created in R^55^ using the R package ggplot2^58^.

**Figure 4:**
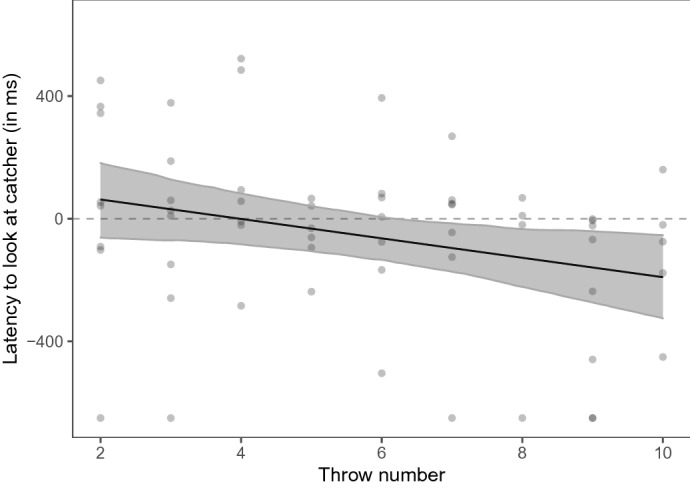
Dogs’ latency to look at the catcher (relative to the video frame before the catcher makes contact with the Frisbee) as a function of the throw number (2–10). The dots depict the individual latencies. The black line depicts the model fit and the grey area around the model fit indicates the 95% confidence interval. The dashed horizontal line highlights the time point right before the Frisbee makes contact with the catcher. Negative values indicate that dogs looked at the catcher before the Frisbee arrived. The figure was created in R^55^ using the R package ggplot2^58^.

We analyzed whether the dogs looked at the catcher before the Frisbee arrived. Therefore, we determined the time point when the dogs first looked at the catcher in a time window of ± 650 ms relative to the video frame before the Frisbee made contact with the catcher (median: − 54 ms; range: − 259 ms–378 ms; see Fig. 3). We fitted a linear mixed model (LMM 01) to examine whether the throw number (2–10) and the freezing of the catcher predicted the dogs’ gaze latency (see Table 1). The dogs looked faster at the catcher with increasing throw number (χ^2^ = 4.81, df = 1, *p* = 0.028). Figure 4 shows that dogs started looking at the catcher before the Frisbee arrived in the second half of the video. Whether the catcher was moving or frozen while the Frisbee moved toward this player did not significantly affect the dogs’ latency to look at the catcher (χ^2^ = 0.17, df = 1, *p* = 0.678).

**Table 1:** Results of LMM 01.

	Estimate	SE	Lower CI	Upper CI	χ^2^	df	p	Min	Max
(Intercept)	− 38.14	53.76	− 133.52	66.33				− 76.29	− 10.13
Catcher movement^1^	− 29.87	70.91	− 174.18	106.33	0.17	1	0.678	− 49.63	− 5.95
Throw number^2^	− 82.89	35.43	− 147.31	− 14.63	4.81	1	0.028	− 102.21	− 60.48

^1^Reference category: frozen; ^2^Throw number was standardized to mean of 0 and a SD of 1. Full-null model comparison: χ^2^ = 5.04, df = 2, *p* = 0.081. min, max: range of estimates obtained when dropping levels of random effects one at a time.

### Experiment 2

In Experiment 2, we froze the entire video four times while the Frisbee was in mid-air (see Supplementary Video S2). We froze the video for the first time on the fourth throw. Then the video was rewound until the Frisbee reached the thrower again. This sequence (throw–freeze–rewind) was repeated once again. Then the video continued normally until the ninth throw when the video was frozen and rewound again for two consecutive times.

**Figure 5:**
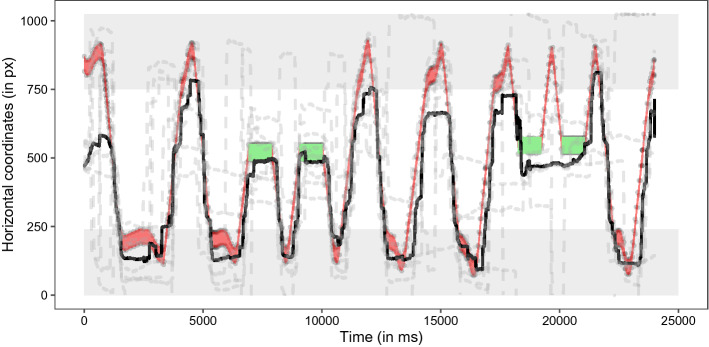
Time series plot showing dogs’ horizontal gaze position across the entire 24-s video in Experiment 2. The dashed grey lines show dogs’ individual performance; the black line shows the median gaze positions; the area highlighted in red shows the position of the Frisbee. The light grey areas highlight the positions of the two players; the green areas indicate when the video was frozen while the Frisbee was hovering in mid-air between the two players. The figure was created in R^55^ using the R package ggplot2^58^.

The movement of the Frisbee again explained a large part of dogs’ horizontal eye movements, though less compared to Experiment 1 (median r^2^ value: 0.42, range: 0.07–0.45; see Fig. 5 and Supplementary Fig. S2 online). To analyze the surprising freezing and rewinding events, we subdivided the video into five interest periods: the first two and last two freeze-rewind sequences and the periods before, in between, and after these sequences. We found that particularly the first two freezing sequences appeared to capture dogs’ attention (first two freeze-rewind sequences: median r^2^ = 0.76; last two freeze-rewind sequences: median r^2^ = 0.11) compared to the unaltered periods before, in between, and after these sequences (beginning: r^2^ = 0.43; middle: r^2^ = 0.45; end: r^2^ = 0.49).

**Figure 6:**
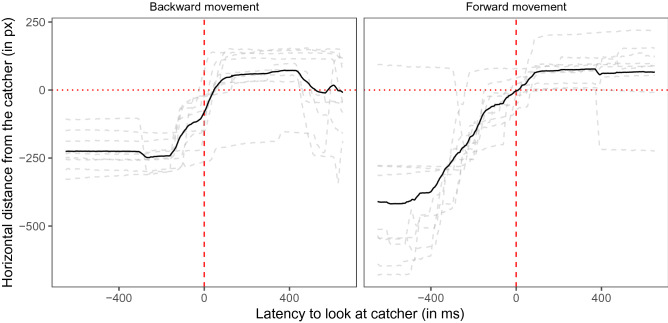
Dogs’ gaze distance from the catcher (positive values are within the catcher AOI, negative values indicate gaze positions between the two players) as a function of the latency relative to the video frame before the Frisbee makes contact with the catcher. The dashed grey lines indicate the median individual latencies; the black line indicates the mean latency (based on the individual median values). The vertical red dashed line indicates the time point right before the Frisbee made contact with the catcher. The horizontal red dotted line highlights the boundary of the catcher AOI. The left panel depicts backward movements of the Frisbee (rewinding) after the Frisbee was frozen in mid-air. The right panel depicts forward movements of the Frisbee. The figure was created in R^55^ using the R package ggplot2^58^.

When the video was frozen the dogs’ gaze sometimes overshot to the next catcher (Dog 4 and 5, see Supplementary Fig. S2). We again analyzed whether the dogs looked at the catcher (including the rewound throwing events) before the Frisbee arrived in the same way as in Experiment 1 (interest period: ± 650 ms relative to the video frame before the Frisbee made contact with the catcher; median: − 19 ms; range: − 173 ms–128 ms). In LMM 02, we examined whether the throw number (2–10) and the backward (rewinding) or forward movement of the Frisbee predicted the dogs’ gaze latency to look at the catcher. Neither the throw number nor the movement direction had a significant effect on dogs’ latency to look at the catcher (see Table 2 and Fig. 6).

**Table 2:** Results of LMM 02.

	Estimate	SE	Lower CI	Upper CI	χ^2^	df	p	Min	Max
(Intercept)	21.67	60.51	− 94.55	131.77				2.07	53.75
Frisbee movement^1^	− 78.69	73.02	− 212.48	72.89	1.15	1	0.28	− 120.88	− 35.70
Throw number^2^	26.74	41.26	− 50.58	106.50	0.41	1	0.52	5.64	57.09

^1^Reference category: backward; ^2^Throw number was standardized to mean of 0 and a SD of 1. Full-null model comparison: χ^2^ = 1.51, df = 2, *p* = 0.471. min, max: range of estimates obtained when dropping levels of random effects one at a time.

The dogs’ reduced tendency to track the Frisbee movement in the second freeze-rewind sequence resulted in many missing observations with respect to the latency to look at the catcher. We therefore subdivided the data into the aforementioned five interest periods (the two freeze-rewind-freeze-rewind sequences and the periods before, in between, and after them) and calculated the mean latencies for each interest period. In LMM 03, we examined whether the Frisbee movement condition (forward or backward) and the interest period number within condition (1–3) predicted the dogs’ gaze latency to look at the catcher. The dogs looked significantly faster at the catcher in the forward movement condition (mean latency: − 103 ms, 95% CI [− 232; − 55]) compared to the backward movement condition (mean latency: 37 ms, 95% CI [− 59; 144]); χ^2^ = 4.22, df = 1, *p* = 0.040; see Table 3). The interest period number within condition had no significant effect on the dogs’ latency to look at the catcher (χ^2^ = 1.30, df = 1, *p* = 0.254).

**Table 3:** Results of LMM 03.

	Estimate	SE	Lower CI	Upper CI	χ^2^	df	p	Min	Max
(Intercept)	41.75	51.90	− 62.92	137.44				25.30	67.37
Frisbee movement^1^	− 182.46	77.72	− 332.06	− 33.38	4.22	1	0.040	− 232.78	− 126.34
Interest period number^2^	60.87	51.56	− 34.05	163.26	1.30	1	0.254	29.80	99.67

^1^Reference category: backward; ^2^Interest period number (within movement condition) was standardized to mean of 0 and a SD of 1. Full-null model comparison: χ^2^ = 5.06, df = 2, *p* = 0.080. min, max: range of estimates obtained when dropping levels of random effects one at a time.

## Discussion

Our results indicate that the dogs accurately tracked horizontal object movements. Over the course of the video shown in Experiment 1, the dogs became faster at looking at the destination of the Frisbee, eventually looking at the catcher before the Frisbee arrived. With increasing experience, the dogs’ motion tracking turned into anticipatory looking. The movements of the catcher did not explain their anticipatory looking. When we froze the whole video in Experiment 2, the dogs maintained their gaze for the most part on the Frisbee. The gaze of two dogs, however, overshot to the next player when the video was frozen for the first time highlighting that these dogs anticipated the destination of the Frisbee and were not merely following the moving object. When comparing the forward and backward Frisbee movement, we found that dogs looked faster at the catcher following forward movements compared to the unexpected backward movements.

In the current study, we used a refresh and video frame rate of 60 Hz. Our results suggest that this rate might be sufficient for motion tracking in dogs. Nevertheless, adopting frame and refresh rates of > 80 Hz might be sensible in future research to ensure that dogs perceive the stimulus movement as continuous motion^6^. The impact of other stimulus properties also deserves further scrutiny in future research including the size, velocity, color, luminance, and contrast of the moving stimuli^2,37^. Additionally, manipulating the identity of the moving object (e.g., running or flying animals and abstract geometric shapes) might be informative to examine how different contexts (e.g., play, hunting) affect motion tracking and anticipation in dogs.

Moreover, the majority of the dogs tested in this study were from herding breeds. To what extent breeds differ in motion tracking and anticipatory looking is another interesting avenue for future research. One might hypothesize, for instance, that sight-hound breeds might be more accurate in motion tracking than terriers whose hunting behavior relies more on olfactory cues^2^. Another interesting aspect might be to what extent brachycephalic and dolichocephalic breeds differ in their motion tracking. The length of the skull across breeds has been found to be correlated with the existence of a pronounced visual streak^38^. The visual streak is a horizontally aligned area on the canine retina with the highest ganglion cell density and therefore, the highest visual acuity (the area centralis exhibits the peak ganglion cell density within the visual streak^3,5^). One might hypothesize that dolichocephalic breeds with a pronounced visual streak might show enhanced motion detection and tracking (at least along the horizontal axis) compared to brachycephalic breeds.

Humans tend to fixate unpredictable objects (for example objects hovering in the air) longer than predictable ones^39,40^. In Experiment 2, the first two freeze-rewind sequences also captured the dogs’ attention as they closely followed the Frisbee location in this period. This attention-grabbing effect, however, appeared to be rather transient. When we presented them with the last two freeze-rewind sequences, the dogs did not closely track the surprising Frisbee movement any longer. The transient nature of this effect might be related to dogs’ weak expectations about gravity^41,42^. Alternatively, the presentation of the visual stimuli on a screen might have attenuated dogs’ reaction to the freeze-rewind event (even though there is evidence that dogs recognize visual content presented on a screen^43^). Freezing videos at different points in time, for example, while an object is hovering in the air and after the object has hit the ground might indicate to what extent dogs’ increased attention is linked to expectations about the physical environment. Previous looking time studies suggest that dogs react to certain violations of physical principles such as size-constancy^44,45^ and solidity^46^. Another possibility to probe the flexibility of dogs’ anticipatory looking might be to present them after some normal trials (videos playing forward) with rewind trials with a reversed playback direction. The dogs would need to learn to anticipate events with a reversed temporal directionality. This approach would show how flexible dogs’ anticipatory looking is and how fast they adapt to new regularities.

A similar method can be used to study the parsing of goal-directed actions. For example, one could interrupt videos either within a goal-directed action sequence or after the completion of a goal-directed action sequence. Human infants looked longer at interruptions within goal-directed actions^47^. Humans also make so-called look-ahead fixations to relevant locations of their own and others’ actions^48–50^. Non-human great apes seem to make similar action predictions^35,36^. It will be an interesting question for future research whether dogs also engage in goal-based action predictions. A recent looking time study indeed suggested that dogs form expectations about goal-directed human actions^51^, which might be the basis for action predictions.

In summary, in the current study dogs accurately followed a moving object on the screen and, with limited experience, began to anticipate its destination. These findings suggest that dogs’ gaze control can be based on predictions of objects’ motion paths acquired through previous experience. We conclude that anticipatory looking and motion tracking paradigms are promising methods for future canine cognition research.

## Methods

### Subjects

In Experiment 1, we tested 14 pet dogs (10 border collies, 3 mixed breeds, and 1 Australian Shepherd; mean age: 8.2 years, range: 4–11 years; 9 females, 5 males). In Experiment 2, we tested 12 pet dogs (8 border collies, 3 mixed breeds, and 1 Australian Shepherd; mean age: 7.6 years, range: 4–10 years; 8 females, 4 males).

### Ethical statement

All experimental protocols were discussed and approved by the institutional ethics and animal welfare committee of the University of Veterinary Medicine, Vienna in accordance with GSP guidelines and national legislation (ETK-39/02/2019). All methods were carried out in accordance with the relevant guidelines and regulations. Informed consent was obtained from all dog owners prior to the study. Informed consent was also obtained for publication of identifying information/images (Fig. 1) in an online open-access publication.

### Stimuli

We presented the dogs with two video clips. The two video clips were based on the same recording depicting how two human players threw a violet Frisbee back and forth (10 times) against a white background (see Fig. 1). In Experiment 1, we froze one of the players while the Frisbee moved toward this player at four different points in the video. The catcher was frozen during throw number 2, 5, 7, and 10. In Experiment 2, we froze the video when the Frisbee was hovering in midair (every time for a period of 1,000 ms) and rewound the video until the Frisbee made contact again with the thrower. We included four of these freeze-rewind sequences in the video.

We showed half of the sample a mirrored version of these videos, i.e. for half of the sample the videos started with the Frisbee on the left, for the other half they started with the Frisbee on the right. The videos had a frame rate of 60 fps and a duration of 16 s (Exp. 1) and 24 s (Exp. 2), respectively.

### Apparatus

We used the EyeLink1000 eye-tracking system (SR Research, Canada) to record the dogs’ eye movements. We sampled the movements of the right eye at 1,000 Hz. We used an adjustable chin rest to facilitate the maintenance of a stable head position during stimulus presentation. We presented the stimuli on a 27-inch LCD monitor (resolution: 1,024 × 768; refresh rate: 60 Hz) at a distance of 50 cm from the dogs’ eyes. The video area subtended visual angles of 48.2 (horizontal) and 30.9 (vertical) degrees. The Frisbee had a diameter of ca. 52 px subtending a visual angle of 2.6°. We adjusted the height of the chin rest and the height and angle of the eye-tracker for each subject. More details concerning the training protocol and the data collection can be found elsewhere^52^.

### Procedure

Dogs first completed a 3-point or 5-point calibration with animated calibration targets (32–50 px) subtending visual angles of 1.6°–2.5° depending on the used target stimulus. We switched from the 3-point to the 5-point calibration over the course of the data collection (5-point calibration: Experiment 1: 5 dogs; Experiment 2: 6 dogs). Following the calibration, we presented a central fixation target (a white expanding circle; max diameter: 90 px; visual angle: 4.5°). The video started once the dogs fixated the target for 100 ms.

### Analysis

We excluded individuals if less than 70% of all fixations fell into the video area (Experiment 1: N = 3; Experiment 2: N = 3) to ensure that our final sample only included dogs that paid sufficient attention to the videos. We analyzed data from 11 (Experiment 1) and 9 dogs (Experiment 2), respectively. We focused in our analysis on the horizontal eye movements because we were interested to what extent the dogs would track or even anticipate the motion of the Frisbee moving between the two players. We did not apply any event-detection algorithms in the current analyses but worked with raw eye movement data. Half of the sample was presented with a mirrored video. For these individuals, we converted the horizontal gaze coordinates back before analyzing the data to bring the entire dataset into the same reference framework.

First, we analyzed to what extent the horizontal location of the Frisbee center predicted variance in dogs horizontal looking pattern. We determined the location of the Frisbee using the dynamic area of interest (AOI) recording function in EyeLink Data Viewer. We measured the Frisbee position on average every 61 ms (Experiment 1) and 55 ms (Experiment 2). We fitted a linear model for each subject with the dogs’ horizontal gaze positions as the response variable and the x-coordinates of the Frisbee center as the predictor variable. We obtained the proportion of variance (r^2^) explained by the horizontal Frisbee position from these models. The models were fitted in R^53^ using the function lm.

For the predictive looking analysis, we analyzed when the dogs first looked at the catcher within periods of ± 650 ms (approximately the time the Frisbee was in the air when thrown between the two players) relative to the video frames before the Frisbee made contact with the catcher (see Fig. 1a). In the entire video, the Frisbee was thrown 10 times between the two players. In Experiment 1, there were 10 interest periods, one for each throw/catching event. In interest periods 2, 5, 7, and 10 the catcher was frozen.

In Experiment 2, the whole video was frozen while the Frisbee was in mid-air and rewound until the Frisbee had reached the previous player again. We analyzed 14 catching events in Experiment 2: four of these interest periods included the rewinding of a throwing event (resembling a catching event).The remaining ten interest periods included the normal catching events (the same ones as in Experiment 1). Additionally, we analyzed five longer interest periods: the first two and last two freeze-rewind sequences and the periods before, in between, and after them.

As response variable, we analyzed the first time the dogs looked at the catcher within each interest period (if they looked at the catcher). For the predictive looking analysis, we excluded the first throw because some dogs tended to look back and forth between the players irrespective of the Frisbee movements at the beginning of the video (see Supplementary Fig. S1). The analysis is based on the observations in which the dogs looked at the catcher in a given interest period (Experiment 1: N = 61 out of 99 catching events; Experiment 2: N = 72 out of 117 catching events). Additionally, we excluded observations in which the dogs had not looked away from the catcher before the current interest period (Experiment 1: N = 1; Experiment 2: N = 3). We fitted linear mixed models (LMM) in R using the function lmer of the package lme4^54^. In Experiment 1 (LMM 01), we included the predictor variables throw number (2–10) and the catcher movement condition (moving, frozen). In Experiment 2, we fitted two models. In LMM 02, we included the predictor variables throw number (2–14) and the Frisbee movement condition (rewind, forward). In LMM 03, we included the predictor variables interest period number (within condition; 1–3) and the Frisbee movement condition (rewind, forward). In all LMMs, we included subject ID as a random effect and all random slope components except for the correlation parameters among random intercepts and random slopes terms^55,56^. Prior to fitting the models, we z-transformed the covariates throw number or interest period number (to a mean of zero and a standard deviation of one). We determined variance inflation factors^57^ for standard linear models excluding the random effects using the R package car. Collinearity was no issue (maximal Variance inflation factor: LMM 01 and 02: 1.00; LMM 03: 1.15). We found no obvious violations of the assumptions of normality and homogeneity of the residuals. We assessed model stability by comparing the estimates derived from the model based on all data with those obtained from models with individual subjects (i.e., the levels of the random effects) excluded one at a time. The models were stable with regard to the fixed effects.

## Supplementary information

Supplementary file1.

Supplementary Video S1.

Supplementary Video S2.

Supplementary Datasets.

## Data Availability

All data analyzed during this study are included in the Supplementary Information files of this article.
