# Supplementary material for: Dogs accurately track a moving object on a screen and anticipate its destination
Source: Sci Rep. 2020 Nov 16;10:19832. doi: 10.1038/s41598-020-72506-5 (PMC7670446; doi:10.1038/s41598-020-72506-5)
Supplement: Supplementary file 1 — Supplementary file1. [file 41598_2020_72506_MOESM1_ESM.docx]

**Dogs accurately track a moving object on a screen and anticipate its destination**

Christoph J. Völter, Sabrina Karl, Ludwig Huber

# Supplementary material

## Video Captions

*Supplementary Video S1* Video showing an exemplar trial of Experiment 1. The dynamic area of interest is shown around the Frisbee. The pink dot indicates the gaze behavior of one of the subjects. On the right side, we added a time series plot showing the horizontal motion path of the Frisbee (grey dashed line) and the horizontal coordinates of the dog’s gaze (in red) for the entire video. The dark grey areas in the plot highlight the periods in the video when the catcher was frozen. The light grey areas in the plot indicate the location of the two players.

*Supplementary Video S2* Video showing an exemplar trial of Experiment 2. The pink dot indicates the gaze behavior of one of the subjects. On the right side, we added a time series plot showing the horizontal motion path of the Frisbee (grey dashed line) and the horizontal coordinates of the dog’s gaze (in red) for the entire video. The green areas in the plot highlight the periods in which the video was frozen. The light grey areas in the plot indicate the location of the two players.

## Experiment 1


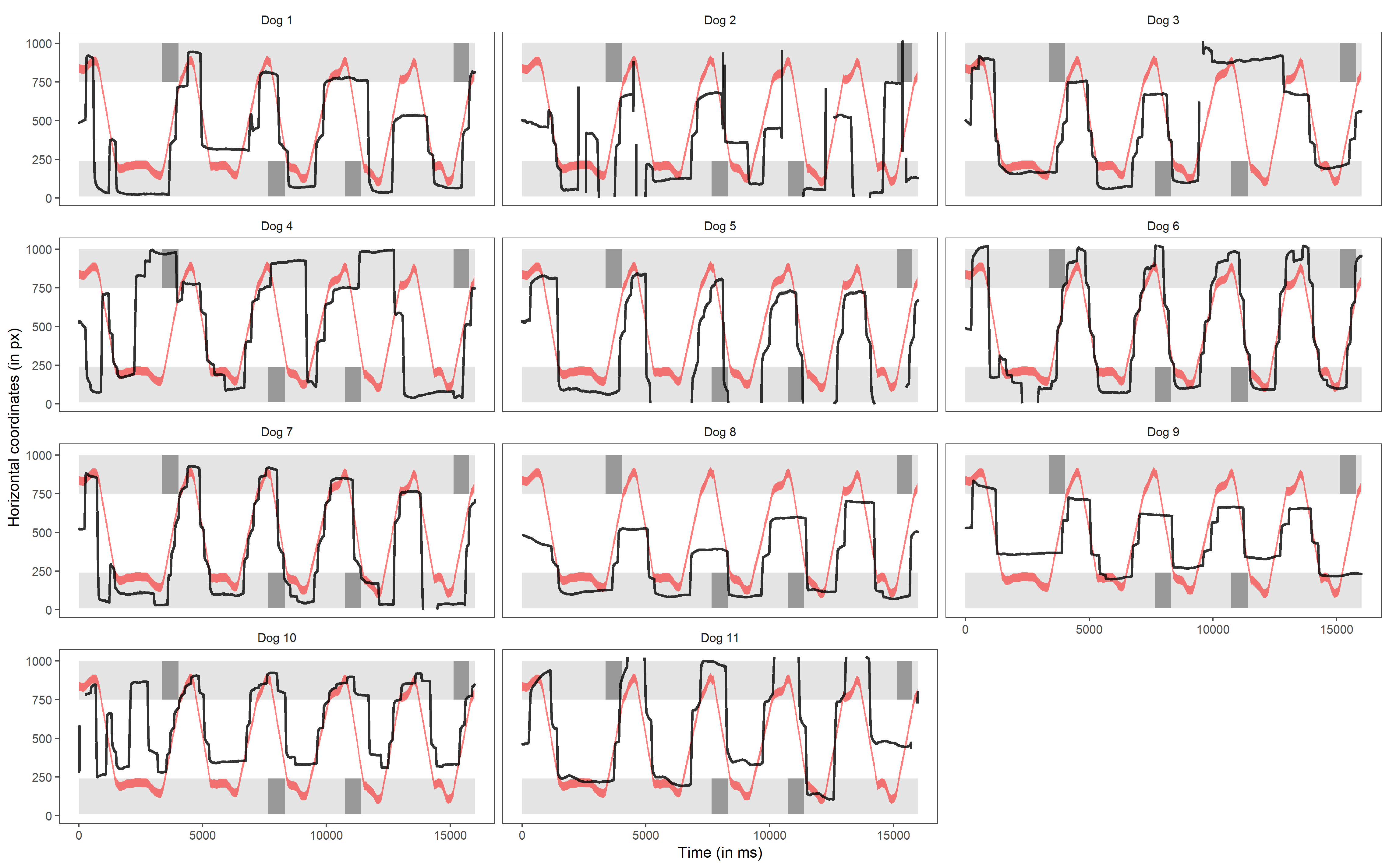


Figure S1 Time series plots showing dogs’ horizontal gaze position (black line) across the entire 16-s video in Experiment 1. The area highlighted in red shows the position of the Frisbee. The light grey areas show the positions of the two players; the dark grey areas highlight when a given player was frozen before the Frisbee arrived.

## Experiment 2


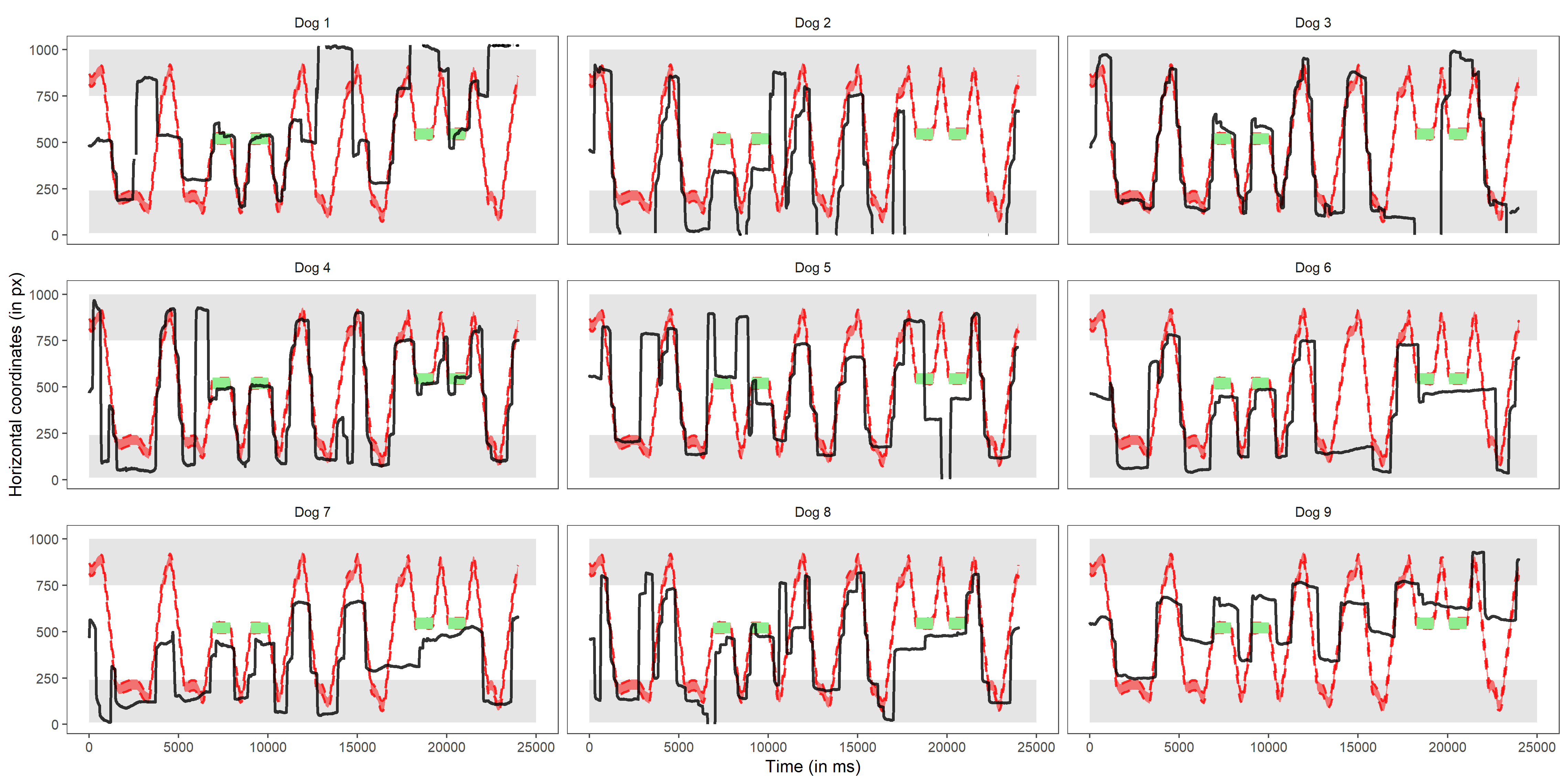


Figure S2 Time series plots showing dogs’ horizontal gaze position (black line) across the entire 24-s video in Experiment 2. The area highlighted in red shows the position of the Frisbee. The light grey areas show the positions of the two players; the green areas highlight when the video was frozen while the Frisbee was hovering in mid-air between the two players.
